# Drowning of Prof. E. Lloyd Howard

**Published:** 1881-09

**Authors:** 


DROWNING OF PROF. E. LLOYD HOWARD.—As we go to press today (Sept. 7th) we are painfully shocked to learn of the drowning on the 5th, of Prof. E. Lloyd Howard, A. M., M. D., by accidentally falling from a tug-boat in the harbor at Baltimore, on his way to the Marine Hospital, of which he was resident physician. He is widely known throughout this country by his superior social, literary, professional and scientific attainments; also for the fearless performance of his professional duties in epidemics. He was President of Md. State Board of Health; Prof. of Chemistry in the Balto. College of Dental Surgery; Prof. of Materia Medica in the College of Physicians and Surgeons of Balto., etc., etc. We know of no one who has accomplished so much at his age (only forty), and we deeply mourn with his family and friends the irreparable loss and his sudden death.

